# Gap Analysis and Conservation Network for Freshwater Wetlands in Central Yangtze Ecoregion

**DOI:** 10.1155/2013/918718

**Published:** 2013-08-25

**Authors:** Li Xiaowen, Zhuge Haijin, Mengdi Li

**Affiliations:** School of Environment and State Key Laboratory of Water Environment Simulation, Beijing Normal University, Beijing 100875, China

## Abstract

The Central Yangtze Ecoregion contains a large area of internationally important freshwater wetlands and supports a huge number of endangered waterbirds; however, these unique wetlands and the biodiversity they support are under the constant threats of human development pressures, and the prevailing conservation strategies generated based on the local scale cannot adequately be used as guidelines for ecoregion-based conservation initiatives for Central Yangtze at the broad scale. This paper aims at establishing and optimizing an ecological network for freshwater wetland conservation in the Central Yangtze Ecoregion based on large-scale gap analysis. A group of focal species and GIS-based extrapolation technique were employed to identify the potential habitats and conservation gaps, and the optimized conservation network was then established by combining existing protective system and identified conservation gaps. Our results show that only 23.49% of the potential habitats of the focal species have been included in the existing nature reserves in the Central Yangtze Ecoregion. To effectively conserve over 80% of the potential habitats for the focal species by optimizing the existing conservation network for the freshwater wetlands in Central Yangtze Ecoregion, it is necessary to establish new wetland nature reserves in 22 county units across Hubei, Anhui, and Jiangxi provinces.

## 1. Introduction

Freshwater ecosystems provide considerable amount of the earth's global biodiversity and substantial ecosystem services, creating a strong imperative for their protection and restoration [1–3]. Although this unique ecosystem has been exposed to higher pressures and threats than adjacent terrestrial ecosystem, freshwater ecosystems have received less attention than terrestrial ecosystems from the conservation community [1, 4–6]. In recent years, freshwater wetlands have internationally received a growing attention due to its globally continuing decline, and freshwater conservation planning has become a newly emerged research field, especially at ecoregional scale [6–13]. However, related case study for freshwater conservation planning is still rare, more research throughout the world is needed to establish scientific conservation strategies for freshwater wetlands worldwide, especially in China where the freshwater ecosystem is unique and diverse globally [8, 14–19].

In the two past decades, gap analysis has emerged in North America as a valuable technique to assist land managers in formulating regional biodiversity conservation planning and building regional conservation networks; numerous gap analysis projects have been developed [1, 20–29]. Gap analysis is also considered to be applicable and valuable in large-scale biodiversity conservation efforts and has been receiving increased attention in China [30]. However, so far there have been few such documented studies for freshwater wetlands at ecoregional scale. 

 The Central Yangtze and its floodplain cover a large area with some of the world's most important and unique freshwater ecosystem, supporting a wide range of important freshwater biotas and associated habitats [31, 32]. Specifically, these habitats act as crucial staging and breeding areas for many globally endangered waterbirds. The Central Yangtze and floodplain currently hosts four Ramsar sites (e.g., Poyang Lake, West Dongting Lake, South Dongting Lake, and East Dongting Lake) and has been designated by the World Wildlife Fund (WWF) as one of the Global 200 Ecoregions (i.e., Central Yangtze Ecoregion), which can be defined as a distinct assemblage of natural communities sharing a large majority of species, dynamics, and environmental conditions that effectively function as a conservation unit [33]. Also, its wetlands play an important role in supplying ecological services, such as microclimate stabilization, flood control, waste and pollutant mitigation, and securing a supply of ground water [34, 35]. Further, the large area of rice growing land in the Central Yangtze provides an essential contribution to regional and national sustainable development needs [34]. 

 In the half past century, the freshwater wetlands in Central Yangtze Ecoregion and the biodiversity they support have been under the constant threat of degradation, mostly associated with human developmental pressures such as large-scale agricultural practices, land reclamation, water and flood control projects, and rapid urbanization [36]. This has resulted in significantly negative consequences, for example, increased flooding, loss of lake and wetland areas, and declines in biodiversity [31, 35, 37–39]. To reverse this trend, the WWF, in collaboration with the Chinese government, has launched a large-scale conservation initiative in Central Yangtze, which is listed as one of the 12 key protection projects in the WWF's global protection network [34]. Following the rapid socioeconomic development, the freshwater wetlands of Central Yangtze have largely fragmented into isolated habitats, and the focus of wetland conservation should therefore shift from those individual-based reserve patterns to the regional conservation network for freshwater wetlands, to ensure the long-term survival of those endangered species and the persistence of these unique freshwater habitats in Central Yangtze Ecoregion.

Due to its internationally important wetlands and globally valuable freshwater biodiversity, the Central Yangtze Ecoregion has already become the target of numerous research projects, including those aimed at detecting changes in habitats and biodiversity, analyzing underlying anthropogenic driving forces, and formulating conservation strategies [31, 37–39]. However, these previous research mainly focused on particular hotspots, such as the Ramsar Sites or nature reserves at a local scale (e.g., Dongting Lake, Poyang Lake, and the Jianghan floodplain, etc.) hence; the conservation strategies were generated based on local and site-based protection and cannot adequately be used as guidelines for a broad-scale conservation initiatives for freshwater wetlands across Central Yangtze Ecoregion. This study therefore aims at establishing a complete and efficient conservation network for freshwater wetlands by refining existing protective system based on a gap analysis of the Central Yangtze, in response to these conservation needs and with a view of ecoregion-based conservation.

## 2. Study Site and Methods

### 2.1. Site Description

**Figure 1:**
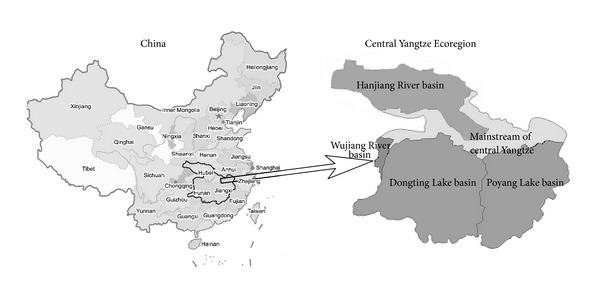
The geographical boundary of Central Yangtze Ecoregion, including its mainstream area and associated large lake and river subcatchments, that is, Dongting Lake Basin, Poyang Lake Basin, Hanjiang River Basin, and Wujiang River Basin, covering an area of 7.55 × 10^5^ km^2^ and accounting for 41.92% of the overall Yangtze River watershed.

WWF delineated the Central Yangtze Ecoregion mainly based on the habitat distribution of focal species (e.g., endangered waterbirds and migratory fish) as well as the ecological integrity of its river and lake basins. Geographically located between E 106°02′–118°36 and N 24°22′–34°16′, the freshwater wetlands of Central Yangtze Ecoregion are mainly composed of some large lake and river subcatchments along its mainstream, for example, Dongting Lake Basin, Poyang Lake Basin, Hanjiang River Basin, and Wujiang River Basin, (Figure 1). Its administrative units include all of Hunan Province, much of Hubei and Jiangxi Provinces, the western part of Anhui Province, and small parts of Fujian, Guangdong, Guangxi, Guizhou, Shanxi, Sichuan, and Zhejiang provinces, covering a total area of 7.55 × 10^5^ km^2^ and accounting for 41.92% of the overall Yangtze River watershed.

### 2.2. Data Source

**Figure 2:**
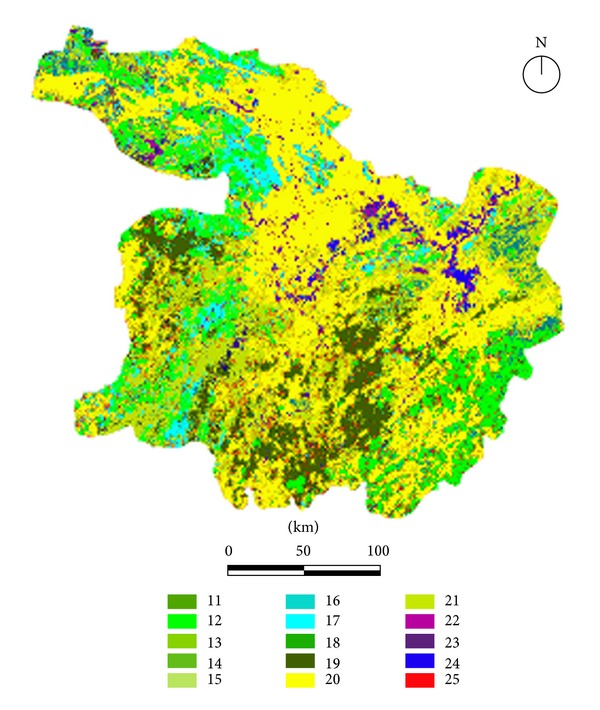
The landuse/landcover data of Central Yangtze Ecoregion obtained and extracted from IGBP 2000 Global landuse/landcover database (11: evergreen needleleaf forest; 12: evergreen broadleaf forest; 13: deciduous needleleaf forest; 14: deciduous broadleaf forest; 15: mixed forest; 16: closed shrub; 17: open shrub; 18: shrub and meadow; 19: grasslands; 20: marsh; 21: cropland; 22: urbanized areas; 23: cropland/natural vegetation mosaic; 24: sparsely vegetated area; 25: surface water).

**Figure 3:**
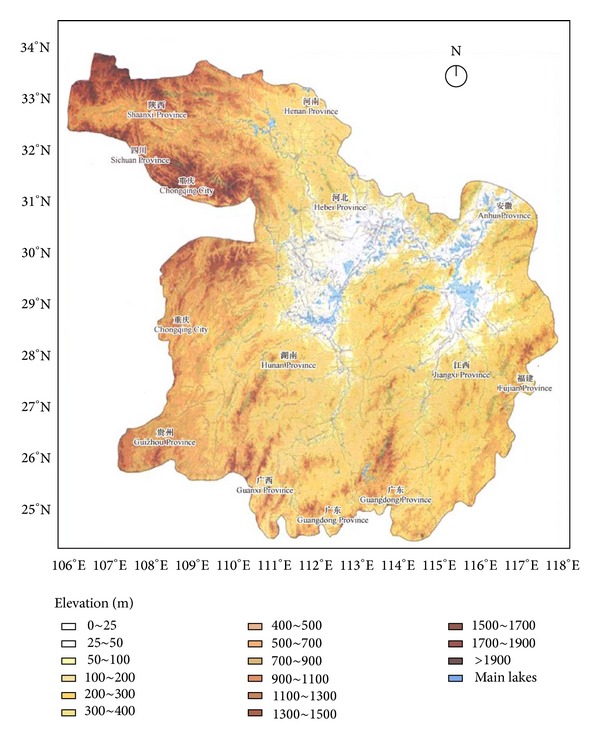
A digital elevation model (DEM) of Central Yangtze Ecoregion derived from SRTM 90m Digital Elevation Database Version 4.1.

The landuse/landcover data were derived from the International Geosphere-Biosphere Programme (IGBP) Global Land cover Database [40] with a grid resolution of 1 km × 1 km (Figure 2). A digital elevation model (DEM) of the Central Yangtze Ecoregion was obtained and extracted from SRTM 90m Digital Elevation Database Version 4.0 [41] (Figure 3), from which the main topographic data (i.e., elevation and slope gradient) were extracted and classified according to their differences in ecological influence based on WWF guidelines (Table 1). Slope gradients were categorized into 6 levels, 0–5°, 5–10°, 15–20°, 20–25°, 25–30°, and >30° (sites with a slope gradient higher than 30° can rarely be used as habitats for the focal species considered here). Elevations were divided into 11 levels, 0–20 m, 20–50 m, 50–100 m, 100–200 m, 200–500 m, 500–1000 m, 1000–1500 m, 1500–2000 m, 2000–2500 m, 2500–3000 m, and >3000 m.

### 2.3. Gap Analysis

To facilitate habitat analysis, the Habitat Suitability Unit (HSU) Index was used to characterize the main habitat types of the focal species. The HSU was defined as the combination of dominant ecogeographical factors affecting habitat suitability (i.e., elevation, slope gradient, and landuse/landcover types). To support the analysis of potential habitats and conservation gaps, a GIS-based spatial dataset of HSUs was built in which a combination of these three factors was used to create a specific HSU type.

**Figure 4:**
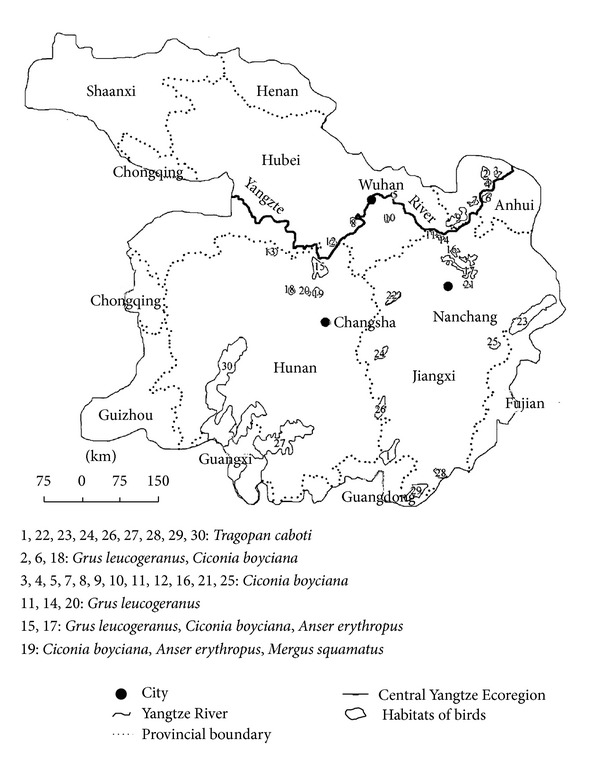
Existing habitats of the focal species in the Central Yangtze Ecoregion identified by the WWF.

 A group of focal species was used in the habitat assessment and gap analysis. The focal species were identified by the following criteria suggested by the WWF and some previous research studies [42, 43]: (1) internationally important species (e.g., species listed in the International Union for Conservation of Nature Red Data Book or famous flagship species that draw considerable public attention); (2) nationally important species, listed in the top or second class of the National Conservation Inventory; (3) endemic species exclusively living in certain areas or habitat types; and (4) umbrella species, whose habitat requirements incorporate the needs of other species. Conserving habitats sufficiently to protect umbrella species can often concurrently protect other species residing within the same ecoregion [43, 44]. Based on these standards, four endangered waterbirds were identified as focal species: Siberian Crane (*Grus leucogeranus*), Oriental White Stork (*Ciconia boyciana*), Lesser White-fronted Goose (*Anser erythropus*), and Chinese Merganser (*Mergus squamatus*). To conduct habitat analysis, the existing habitat distribution data of the focal species was identified by WWF experts in a series specific symposiums organized by WWF in China in 2004 (Figure 4).

 The habitat analysis was based on the hypothesis that if field records indicated that a focal species could be found within a certain area, then the dominant combinations of ecogeographic factors (i.e., elevation, slope gradients, and landuse/landcover types) would constitute the main types of HSU preferred by the focal species. Through overlaying the spatial data of the ecogeographic factors using GIS, the spatial linkages between the HSUs and the focal species were thus built by detecting and identifying the dominant HSUs for each focal species within their existing habitats. These main HSU types of the focal species were then further screened and refined based on their detailed habitat information obtained from the literature review and expert consultation to minimize errors in the analysis. In this manipulation, a large variety of combinations of ecogeographic factors were produced, but most were deleted from the HSU spatial dataset because of insignificant spatial linkages to the habitats as demonstrated by their minor area contribution to the existing habitats. Here, considering the concept of minimum critical area for the long-term survival of focal species, we chose HSU types from these combinations of ecogeographic factors based on the criteria that their area contribution should exceed 5% of the total existing habitat area.

**Figure 5:**
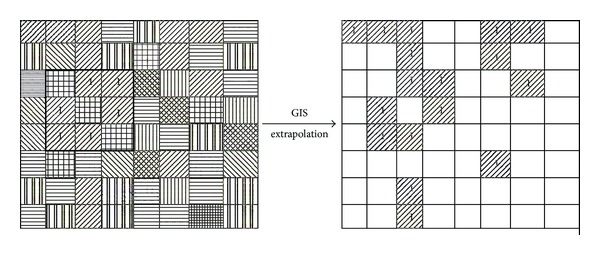
The sketch map to analyze the potential habitats of the focal species.

 The key objective of gap analysis is identifying those potential habitats (i.e., biodiversity hotspots) which have been excluded from existing conservation systems. To analyze potential habitats, we supposed that if a habitat of a focal species can be represented by a group of HSUs, then other unsurveyed habitats sharing the same HSUs within a certain ecoregion can be considered the potential habitats for the focal species. Accordingly, potential habitats can be extrapolated and predicted at a larger scale by GIS-based spatial linkage between the species and the associated types of HSU (Figure 5). To verify the predicted results of the potential habitats, the existing status of the wetlands in those counties with unprotected potential habitats was examined through a literature review and the latest monitoring data from the State Forestry Administration of China.

**Figure 6:**
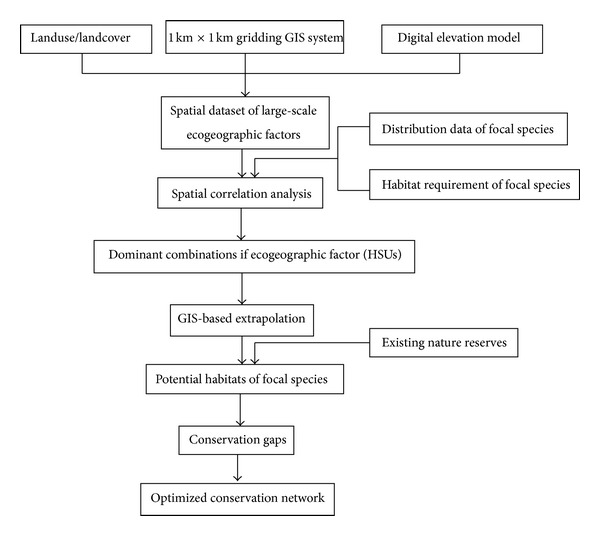
The flow chart to develop optimized wetland conservation network in Central Yangtze Ecoregion.

 Based on the above potential habitat analysis, conservation gaps (i.e., unprotected potential habitats) were located by comparing protected areas (i.e., existing nature reserves) with potential habitats. Then, the conservation network of the Central Yangtze Ecoregion could be established by combining the conservation gaps with the existing conservation system (Figure 6).

 Because precise spatial data of the nature reserves is lacking (especially at provincial and local levels), the county units were therefore used as the basic spatial units for the gap analysis and conservation network planning in our research. We assumed if only one county has at least one wetland nature reserve, then the freshwater wetlands and the associated biodiversity in the county could be effectively protected.

## 3. Results

The results of habitat analysis showed that the surveyed distribution area of Siberian Crane is 2201.4 km^2^, including 33 types of ecogeographic combinations, of which four types were identified as HSUs with total area contribution of 81.1% to the potential habitat, including the types of 11125 (44.2%), 12123 (16.4%), 12125 (11.8%), and 11123 (8.7%). 45 types of ecogeographic combinations of oriental white stork were generated from its surveyed distribution area (3567.1 km^2^), of which four types, that is, 11125, 12123, 11123, and 12125 were extracted as HSUs, accounting for 49.2%, 14.6%, 9.4%, and 9.1% of the potential habitat, respectively, and totally contributed 82.3% to the potential habitat. With regard to lesser white-fronted goose, there are 20 ecogeographic types within its distribution area (1761.5 km^2^), of which five types were extracted as HSUs (i.e., 11125, 12123, 12125, 11123, and 11117), accounting for 49.9%, 14.8%, 12.7%, 6.7%, and 6.6% of the potential habitat, respectively, and totally contributed 90.7% to the potential habitat. With the smallest distribution area of 161.2 km^2^, Chinese Merganser had only 13 ecogeographic types, of which the 6 types were recognized as HSUs (i.e., 11123, 11125, 11119, 12123, 11117, and 12118), accounting for 24.2%, 19.9%, 16.1%, 11.8%, 10.6%, and 5.0% of the potential habitat, individually, and totally contributed 87.6% to the potential habitat.

**Figure 7:**
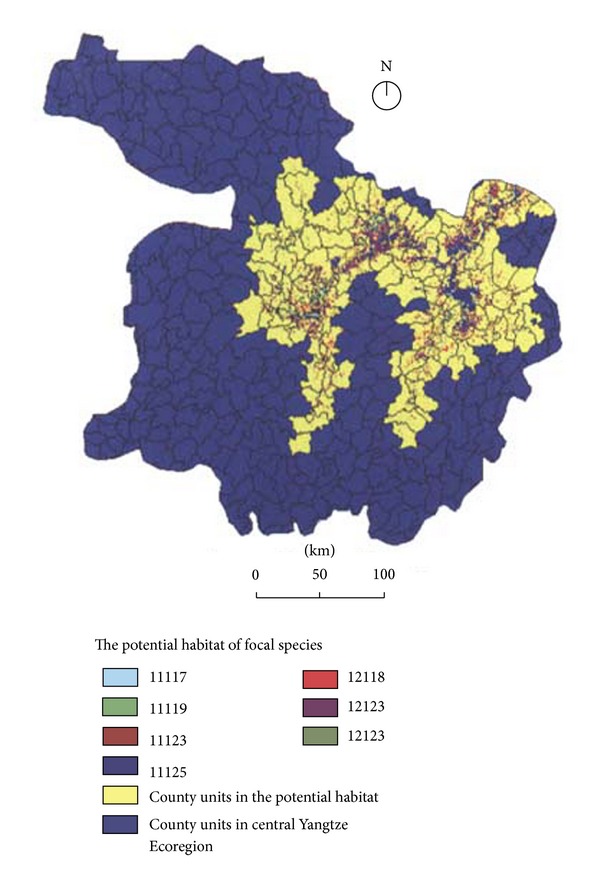
Potential habitats of focal species in the Central Yangtze Ecoregion. All potential habitats cover an area of 32,050 km^2^ and are mainly composed of seven types of Habitat Suitability Units (HSUs), including 132 county units of four provinces, for example, Hubei, Hunan, Jiangxi, and Anhui provinces.

 Through habitat analysis for the focal species, seven HSU types were identified from the large varieties of combinations of ecogeographic factors, 11125, 12123, 12125, 11123, 11119, 11117, and 12118, accounting for 84.0% of the overall potential habitat area (Figure 7 and Table 1 explain these codes for HSUs). The results revealed the core potential habitats represented by the dominant HSUs (i.e., 11125 and 12125) are characterized by surface water in the Central Yangtze floodplain, which occupies 47.30% of the total potential habitats and is mainly made up of shallow wetlands of the main lakes (e.g., Dongting Lake, Poyang Lake and Honghu Lake) in the Central Yangtze Ecoregion. Various waterbirds prefer these HSUs and core potential habitats as foraging and resting habitats. Areas dominated by sedges, meadows, and open shrubs (i.e., 11119, 11118, and 11117) form secondarily important HSUs of potential habitats, occupying 26.68% of the total potential habitats. These HSUs are especially preferred by wintering waterbirds as their core habitats. Also, the ecotones between croplands and natural vegetation (i.e., 11123 and 12123) function as complimentary potential habitats for focal species; in particular, they provide important forging habitats for some endangered large wading birds, such as white crane and oriental white stork. The overall potential habitats of these dominant HSUs include parts of 132 county units with a total area of 32,050 km^2^. Of these, the top four counties rich in potential habitats are Wuchang (1575 km^2^, Hubei Prov.), Susong (1476 km^2^, Anhui Prov.), Jinxian (1217 km^2^, Jiangxi Prov.), and Poyang (1143 km^2^, Jiangxi Prov.), which account for 78.4%, 61.7%, 62.3%, and 27.1% for the administrative areas of these counties and contribute 4.9%, 4.6%, 3.8%, and 3.6% to the total potential habitats, respectively.

Currently, 16 wetland nature reserves have been established in the Central Yangtze Ecoregion, which incorporate the most ecologically valuable parts of the potential wetland habitats, with a total area of 7530 km^2^, including national nature reserves (NNRs) such as the Dongting Lake NNR and the Poyang Lake NNR. However, our analysis revealed that most of potential habitats are still exposed to human impacts, of which only 23.49% was included into the existing wetland nature reserves. Also, the existing conservation pattern presented by the county units seems disorganized and fragmented and can hardly provide a long-term and large-scale conservation in Central Yangtze. The result underscores the urgent need to optimize the existing conservation pattern by filling the conservation gaps and establishing a conservation network in Central Yangtze.

 The significant conservation gaps for freshwater wetlands in Central Yangtze could be identified in counties rich in potential habitats but unprotected by existing nature reserves. These conservation gaps were further refined based on the following criteria: (1) unprotected potential habitats adjacent to existing nature reserves should be selected as gaps, as existing nature reserves act as the core habitats in the Central Yangtze Ecoregion, (2) the selected gaps should be ecologically integrated with existing nature reserves so as to form an optimized conservation network with an interlinked conservation pattern, and (3) those county units sharing a larger proportion of potential habitats should be given priority so that the conservation network can provide effective protection for the potential habitats with the least land cost. 

**Figure 8:**
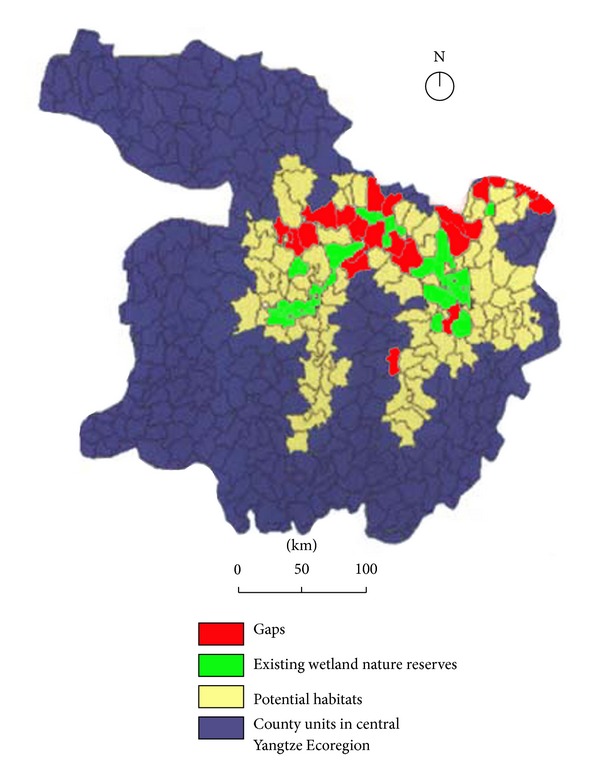
Existing wetland nature reserves, potential habitats, and conservation gaps in the Central Yangtze Ecoregion. The conservation gaps were identified by comparing potential habitats with existing wetland nature reserves, while optimized conservation network can be established by combining the existing nature reserves with the proposed nature reserves based on the gap analysis.

 After screening, 22 county units were categorized as conservation gaps, including 13 in Hubei Province, eight in Anhui Province, and one county of Jiangxi Province (Figure 8 and Table 2). Thus, the combination of existing (23 county units) and proposed (22 county units) conservation systems constitutes an ecologically optimized conservation network for freshwater wetlands in the Central Yangtze Ecoregion, which can be expected to effectively conserve 84% of total potential habitats of focal species in Central Yangtze Ecoregion.

## 4. Conclusions

Our research indicated that a number of wetland nature reserves have been established in the Central Yangtze, but the existing wetland nature reserve system is still far from being effective in conserving the freshwater biodiversity represented by the focal species in Central Yangtze Ecoregion. Our habitat analysis shows that the potential habitats for the focal species in the Central Yangtze Ecoregion include parts of 134 county units, of which the existing wetland conservation system only covers 23.49%. Large parts of these potential habitats are not included in the current protection system and are exposed to human activities such as agricultural development, hydrological projects, and urbanization. Moreover, the existing conservation areas are fragmented and isolated from each other, and so the existing conservation system in Central Yangtze must be adjusted and optimized.

In consideration of maximized representativeness (e.g., proportion of potential habitats) and connectivity of the conservation system with minimized land cost, the optimized conservation network for the freshwater wetlands in Central Yangtze Ecoregion could be established by integrating the existing wetland conservation system with the identified conservation gaps. This optimized wetland conservation network would effectively protect over 80% of the potential habitats for the focal species in Central Yangtze Ecoregion. It would comprise 45 county units across the Central Yangtze, of which 22 would need to establish new protected areas to fill their conservation gaps, including 13 counties in Hubei Province, eight in Anhui Province, and one county in Jiangxi Province.

 In our research, although the accuracy of the current habitat analysis may have been restricted by the resolution (1 km × 1 km) of the landuse/landcover classification due to the hugescale of the research area, the potential habitats revealed by the research, especially those counties with larger proportion of conservation gaps, can be explained and verified by existing documentation. Also, our results show that all of the existing wetland natural reserves were included in the identified potential habitats, indicating that it is applicable to employ Habitat Suitability Units (HSU) and GIS-based habitat extrapolation in large-scale gap analysis.

## Figures and Tables

**Table 1:** Grading and coding the habitat suitability factors of elevation, slope, and landuse/landcover types. Codes of habitat suitability are composed of elevation, slope, and landuse/landcover and are designed to facilitate grid-based operations in GIS. For example, the code 11125 represents an area of habitat with an elevation code of 11 (i.e., 0–20 m), a slope code of 1 (0–5°), and the landuse/landcover code of 25 (i.e., surface water).

Elevation (m)	Code	Slope	Code	Landuse/landcover type	Code
0–20	11	0–5	1	Evergreen needleleaf forest	11
20–50	12	5–10	2	Evergreen broadleaf forest	12
50–100	13	10–15	3	Deciduous needleleaf forest	13
100–200	14	15–20	4	Deciduous broadleaf forest	14
200–500	15	20–25	5	Mixed forest	15
500–1000	16	25–30	6	Closed shrub	16
1000–1500	17	30–35	7	Open shrub	17
1500–2000	18			Shrub and meadow	18
2000–2500	19			Grasslands	19
2500–3000	20			Marsh	20
>3000	21			Cropland	21
				Urbanized area	22
				Cropland/natural vegetation mosaic	23
				Sparsely vegetated area	24
				Surface water	25

**Table 2:** Conservation gaps of wetlands in the Central Yangtze Ecoregion.

No.	County	Wetland list	Province
1	Tongling	Dayehu lake	Anhui
2	Tongcheng	Caizihu lake	Anhui
3	Congyang	Lakes of Baidanghu, Chengyaohu, and Caizihu	Anhui
4	Taihu	Hualiangting reservoir	Anhui
5	Susong	Pohu lake	Anhui
6	Wangjiang	Lakes of Wuchanghu and Pohu	Anhui
7	Lujiang	Lakes of Caohu and Huangpihu	Anhui
8	Wuwei	Caohu lake	Anhui
9	Nanchang	Jiang'an Valley Wetlands	Jiangxi
10	Daye	Dayehu lake	Hubei
11	Wuchang	Lakes of Qingxunhu, Luhu, and Futouhu	Hubei
12	Huangpi	Wuhu lake	Hubei
13	Xinzhou	Lakes of Wuhu and Zhangduhu	Hubei
14	Shashi	Hujiang Wetlands	Hubei
15	Hanchuan	Chahu lake	Hubei
16	Chibi	Huanggaihu lake	Hubei
17	Jiayu	Xilianghu lake	Hubei
18	Yangxin	Lakes of Wanghu and Dayehu	Hubei
19	Xiantao	Paihu lake	Hubei
20	Tianmen	Chahu lake	Hubei
21	Qianjiang	Changhu lake	Hubei
22	Jiangling	Changhu lake	Hubei
